# Supplementary material for: Do we agree on who is playing the ball? Developing a video-based measurement for Shared Mental Models in tennis doubles
Source: PLoS One. 2020 Dec 1;15(12):e0242783. doi: 10.1371/journal.pone.0242783 (PMC7707568; doi:10.1371/journal.pone.0242783)
Supplement: Attachment — Submitted filename: Response to Reviewers.docx [file pone.0242783.s001.docx]

**Reviewer #1: I commend the authors for a well done review and an interesting paper that has certainly contribute to the literature.**

*Thank you so much! That is so nice to hear and makes us happy.*

**Reviewer #2: I acknowledge the response to my comments and the changes made by authors. The manuscript, in the current form, is near to be accepted.
Only remain an aspect that I consider that should be improved.
This aspect is related to the qualitative assessment of the pilot study. As I mentioned previously, I think that the assessment and data analysis is not accurate and still vague and not enough objective. Also, I was concerned about the effect of this qualitative assessment to the main experiment. The qualitative assessment highlight three main critiques. In this way, how authors modified the pilot study based on these critiques? Another question is related to the first critique made by the players, where players stated warm-up as a positive issue. Why is it considered as a critique?

Maybe if a quotation of a player is representative of each criticism it could be added to the manuscript.

overall, I have to congratulate authors by their good job.**

*First, thank you so much, for the compliments. We are really happy to hear that, however, we see your concern and we are sorry, that our changes last time weren’t enough.*

*Due to the length of the whole manuscript we previously shorten that point. However, to address your comment and analyse this section more objectively we looked at the descriptives of how many participants reported that. We included this in the data analysis section. We furthermore, removed the critique with the warm-up videos. As we now state to focus only on problems. Furthermore, we changed the title of “feasibility” to “feasibility feedback” in order to prevent misleading the reader.*

*(Data Analysis Section (line 312-316)*

The feasibility of the study was identified through asking unstandardized open questions for feedback of the participants after completion of the study and to check whether problems emerged. Answers were grouped together with unstandardized observations of the experimenter during the pilot study. Problems which emerged half of the time or more were reported.

(Results Section line 342 - 348)

Feasibility Feedback

In general, participants found the study feasible, however two problems emerged: First, participants reported to have difficulty maintaining a high level of concentration throughout all four conditions and reported getting tired after a while. Second, participants noticed that the videos remained the same in every condition. Some were even able to recall their own decisions on previous videos and this influenced their decisions on the videos in the later conditions.

***Modification based of the pilot study, based on the critiques***

*Based on the feedback of the participants, we described the modification in the interim discussion (see line: 361ff):*

“However, based on the feedback about the difficulty in concentrating and, even more importantly, on recalling their own decision on prior videos, we decided to shorten the video measurement. First, we decided to only measure the SMM for the net player and thus, using only two conditions. Second, as the main study should measure more intuitive decisions rather than deliberate ones, we added a 3-s time limit for ball-taking behavior and movement directions to avoid participants being able to recall their actions and deliberately thinking about their decisions.(cf. [46]). “
